# Fetus in Fetu as a suprarenal mass in a neonate – a rare and perplexing entity

**DOI:** 10.4322/acr.2021.347

**Published:** 2022-01-04

**Authors:** Namratha Ravishankar, Sheeladevi CS, Joxce Pazhayattil

**Affiliations:** 1 JSS Medical College, JSS Academy of Higher Education and Research, Department of Pathology, Mysore, India

**Keywords:** Biopsy, fine needle, Adrenal Glands, Teratoma, Fetus

## Abstract

Fetus in fetu (FIF) is a rare entity in which a malformed vertebrate fetus is incorporated within its twin. This entity should be differentiated from a teratoma, which has more malignant potential. We describe a neonate with a heterogeneous calcified suprarenal mass. The aspiration cytology revealed a heterogeneous cell population with spindle cells and small round blue cells. Operative and histopathologic examination showed features consistent with a fetus in fetu. This case report describes a rare entity and discusses its clinical presentation, cytological features on fine-needle aspiration, and the difficulties posed in its differentiation from a teratoma.

## INTRODUCTION

Fetus-in-fetu (FIF) is an uncommon anomaly of embryogenesis with an incidence of 1 per 500,000 births in which a vertebrate fetus is incorporated within the body of its twin. 1

Only around 200 cases of FIF have been reported in literature, and the most common clinical presentation is an abdominal mass in a young infant.^2^ A conjectural diagnosis can be made by plain radiography, ultrasonography, computed tomography( CT), or magnetic resonance imaging (MRI), and occasional cases are even diagnosed during prenatal screening. 3 The entity’s most common site is in the retroperitoneum (80%), but it can also be found in the skull, oropharynx, left ventricle, sacrum, ovary, and testis.^4^ Complete excision of the mass is curative, and histopathology is essential for the diag nosis of FIF. The presence of a vertebral column is crucial in differentiating it from a teratoma. This differentiation is vital due to the malignant potential of the latter, although rare cases of FIF with malignant recurrence have been reported.^5^ Another diagnostic modality by molecular analysis is using an informative genetic marker for uniparental isodisomy of chromosomes 14 and 15. If there is no genetic difference between the host and the fetiform mass, then it is diagnostic of FIF.^3^ Owing to its uncommonness, FIF is not usually considered as the first possibility in a neonate with an asymptomatic abdominal mass. This case of FIF is reported for its rarity and to discuss the limitations of making a diagnosis of FIF on aspiration cytology as well the difficulties encountered in its differentiation from a teratoma. The pathologist identified the noncalcified vertebral column invisible on the radiographs; therefore, the nonvisualization of the vertebral axis on radiography or on computed tomography scan does not exclude the diagnosis of fetus in fetu.

## CASE REPORT

**Figure 1:**
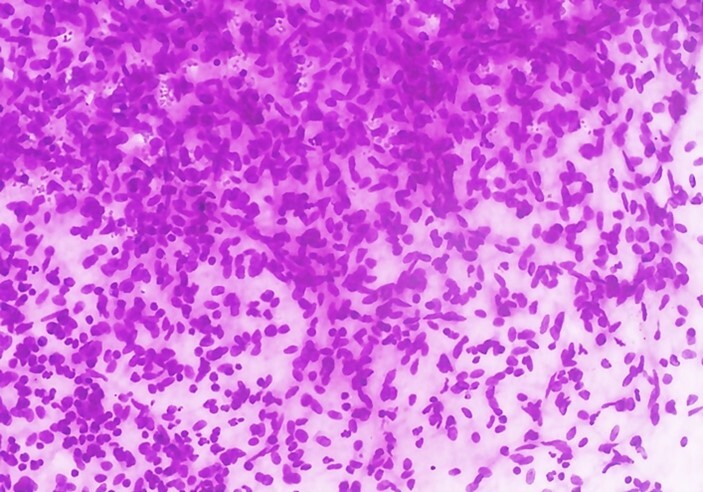
FNA cytology of Fetus in fetu showing small round blue cells and spindle-shaped cells (H&E, 100x).

A one-month-old female baby was referred to a tertiary care center as antenatal ultrasonography had revealed a right suprarenal mass in the fetus in the third trimester. On presentation, the baby was feeding well, passing stools, and had no complaints. On examination, the abdomen was soft, non-tender and no palpable mass was found. Echocardiography revealed a congenital heart disease with large ostium secundum ASD. The ultrasound evaluation demonstrated a well-defined lobulated heterogeneous mass measuring 6.2x4.4x5.6 cm in the right suprarenal region, predominantly hyperechoic with multiple cystic areas and internal vascularity. There was no family history of twin births. The mass was aspirated under ultrasound guidance, and paucicellular smears were obtained. The aspirates showed loose aggregates of small round cells, spindle-shaped cells, and scattered ganglion cells (Figure 1). The probable differential diagnoses of neuroblastoma and teratoma were considered.

**Figure 2:**
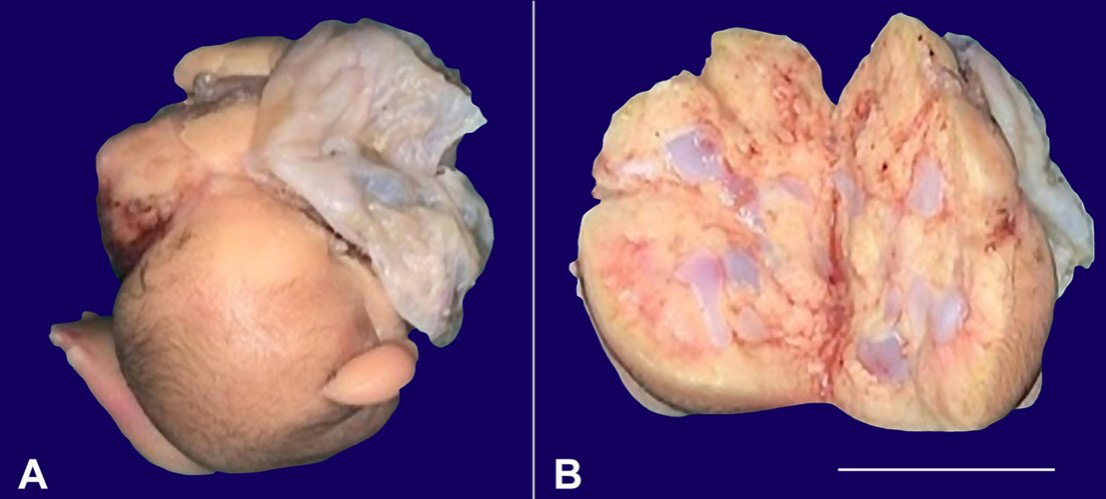
**A** and **B** – Resected fetiform mass covered with skin with cartilage on cut section with an attached membrane (scale bar 5 cm).

The baby underwent laparotomy, and per-operatively, the renal and adrenal vessels were identified supplying the mass, which was removed en bloc. A single irregular fetiform mass was seen within a sac, covered with skin measuring 7.5 x 4.8 x 4.5 cm with attached membranes and rudimentary limb buds. The cut surface was grey, yellow with cartilaginous areas (Figure 2).

**Figure 3:**
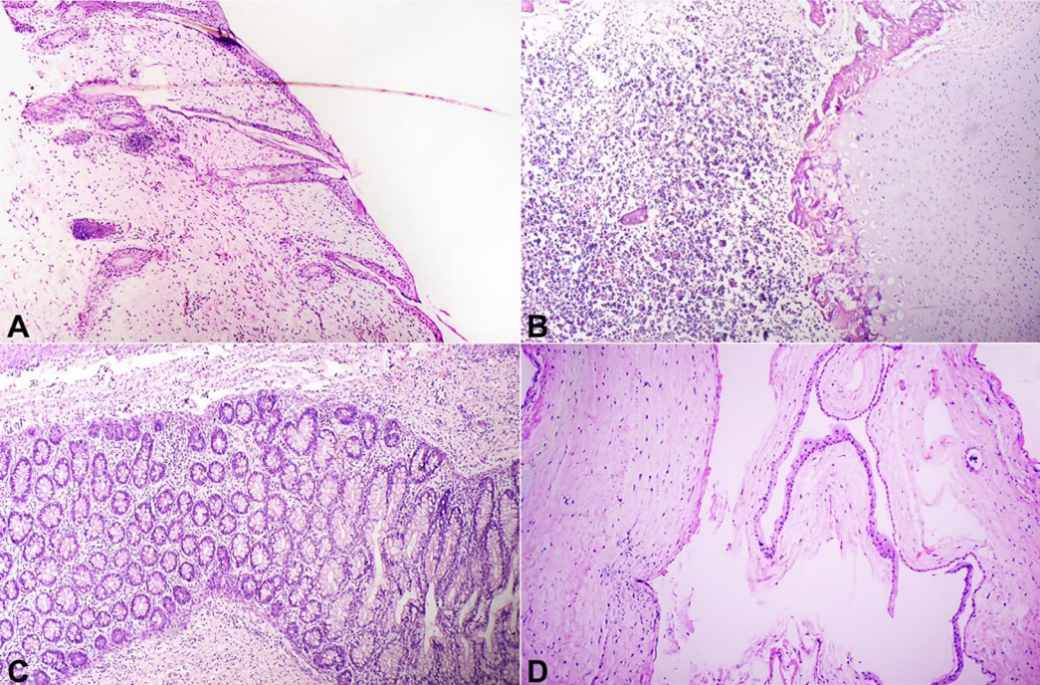
Photomicrographs of fetiform mass showing: **A** – skin with adnexa (H&E, 100x); **B** – structure resembling vertebra composed of bone marrow, cartilage, and bone (H&E; 100x); **C** – gut layer lined by colonic mucosa (H&E, 100x); **D** – structure resembling membrane showing chorion and amnion (H&E, 100x).

Histopathologic examination of the fetus revealed skin with adnexa, a vertebral column with cartilage, bone and bone marrow elements, striated muscle, sympathetic ganglia, adipose tissue, and parts of the gastrointestinal tract consisting of stomach, small and large intestine (Figure 3).

Histopathological examination of the resected membrane was consistent with a fetal sac containing chorion and amnion. There were no immature components or features of malignancy seen. The post-operative period was uneventful. At the last follow-up, the baby had no complaints.

## DISCUSSION

The term fetus in fetu was first introduced by Friedrich Meckel^6^ in the eighteenth century.

The exact embryogenesis of FIF is mired in controversy, and the most accepted hypothesis is the included twin theory, which asserts that it is an anomaly of monozygotic diamniotic twinning where the unequal division of the totipotent inner cell mass leads to the assimilation of the smaller cell mass within the maturing twin embryo following anastomosis of the vitelline circulation.^7^ The other hypothesis is the teratoma theory, where FIF is regarded as a highly differentiated form of a mature teratoma.^8^

The most common site of FIF is the retroperitoneum, and other sites include the CNS, oral cavity, neck, adrenal gland, scrotum, pelvis, and mediastinum.^8^ To be diagnosed as a FIF, one of the following criteria must be fulfilled: a mass within a distinct sac, partially or completed covered by skin, macroscopically evident anatomic features, attachment to host by a pedicle containing a few large blood vessels, and located immediately adjacent to one of the sites of attachment of conjoined twins or associated with the neural tube or the GIT.^3^

The presence of a vertebral column is the defining feature that distinguishes this entity from a teratoma whose development does not advance beyond the primitive streak stage. This differentiation is of paramount importance because of the malignant potential of teratomas. In contrast, FIF is almost always benign, and only an occasional case of malignant FIF has been reported in -literature.^8^^,^ 9 The vertebral column is undetectable even on microscopic examination in 9% of cases of FIF, and this has garnered support for the teratoma theory of FIF. However, complete excision of FIF along with the sac is essential due to the possibility of malignant recurrence if the tissue is not completely excised.^9^

FIF is thought to develop in a manner analogous to the normal development of a fetus, while teratomas consist of pluripotent cells, with no clear-cut organogenesis or segmentation of the vertebral column.^10^ Imaging plays a pivotal role in making a preoperative diagnosis of FIF which is a differential diagnosis in a newborn with a complex abdominal mass with calcific components.^5^ The other possibilities to be considered in this scenario include teratoma, meconium pseudocyst, and neuroblastoma.^8^

Fine needle aspiration cytology features of FIF have been discussed only in very few scenarios where it mimicked teratoma and teratoid Wilms tumor cytologically owing to the presence of a heterogeneous cell population.^11^ In our case, too, the presence of both spindle cells and small round cells prompted us to consider differential diagnoses of teratoma and neuroblastoma. This is highlighted as a pitfall in the diagnosis of teratoid lesions of childhood on FNA cytology, where supplementary radiologic investigations are instrumental in arriving at the correct diagnosis.^11^

## CONCLUSION

FIF should be contemplated as an important differential diagnosis in a neonate or an infant with a calcified abdominal mass. The preoperative diagnosis of FIF is based on a vertebral column or limbs on radiological imaging, and many cases are diagnosed early during prenatal obstetric ultrasonography. While imaging modalities and FNAC of the mass may prove fruitful, they lack specificity as in our patient, which warrants excision and histopathological examination. The vertebral column which may be undetectable on radiographs can be identified by the pathologist and histopathology is instrumental in differentiating it from a teratoma. Current guidelines emphasize the importance of differentiating a teratoma from FIF due to the 10% malignancy rate in teratomas. Although FIF is considered a benign entity, the rare possibility of malignancy in FIF warrants close clinical, radiological, and serological follow up.
